# Cefepime dosing regimens in critically ill patients receiving continuous renal replacement therapy: a Monte Carlo simulation study

**DOI:** 10.1186/s40560-018-0330-8

**Published:** 2018-09-12

**Authors:** Weerachai Chaijamorn, Taniya Charoensareerat, Nattachai Srisawat, Sutthiporn Pattharachayakul, Apinya Boonpeng

**Affiliations:** 1 Faculty of Pharmacy, Siam University, 38 Petkasem Road, Bangwa, Pasicharoen, Bangkok, 10160 Thailand; 2 Division of Nephrology, Department of Medicine, Faculty of Medicine, Chulalongkorn University, and King Chulalongkorn Memorial Hospital, Bangkok, Thailand; 3 Department of Clinical Pharmacy, Faculty of Pharmaceutical Sciences, Prince of Songkla University, Songkhla, Thailand; 4 School of Pharmaceutical Sciences, University of Phayao, Phayao, Thailand

**Keywords:** Cefepime, Dosing, Pharmacokinetics, Pharmacodynamics, Continuous renal replacement therapy, Critically ill patients

## Abstract

**Background:**

Cefepime can be removed by continuous renal replacement therapy (CRRT) due to its pharmacokinetics. The purpose of this study is to define the optimal cefepime dosing regimens for critically ill patients receiving CRRT using Monte Carlo simulations (MCS).

**Methods:**

The CRRT models of cefepime disposition during 48 h with different effluent rates were developed using published pharmacokinetic parameters, patient demographic data, and CRRT settings. Pharmacodynamic target was the cumulative percentage of a 48-h period of at least 70% that free cefepime concentration exceeds the four times susceptible breakpoint of *Pseudomonas aeruginosa* (minimum inhibitory concentration, MIC of 8). All recommended dosing regimens from available clinical resources were evaluated for the probability of target attainment (PTA) using MCS to generate drug disposition in a group of 5000 virtual patients for each dose. The optimal doses were defined as achieving the PTA at least 90% of virtual patients with lowest daily doses and the acceptable risk of neurotoxicity.

**Results:**

Optimal cefepime doses in critically ill patients receiving CRRT with Kidney Disease: Improving Global Outcomes (KDIGO) recommended effluent rates were a regimen of 2 g loading dose followed by 1.5–1.75 g every 8 h for Gram-negative infections with a neurotoxicity risk of < 17%. Cefepime dosing regimens from this study were considerably higher than the recommended doses from clinical resources.

**Conclusion:**

All recommended dosing regimens for patients receiving CRRT from available clinical resources failed to achieve the PTA target. The optimal dosing regimens were suggested based on CRRT modalities, MIC values, and different effluent rates. Clinical validation is warranted.

## Background

Continuous renal replacement therapy (CRRT) is generally performed in hemodynamic unstable patients with acute kidney injury (AKI) [1]. Cefepime is an antimicrobial agent that is commonly used in critically ill patients. The low protein binding affinity (16–20%) and small volume of distribution (14–20 L) make cefepime susceptible to be removed by CRRT [2–4].

Pharmacokinetic changes in critically ill patients, such as increasing of volume of distribution and hypoalbuminemia, considerably reduce hydrophilic antimicrobial agent concentrations [5]. Consequently, we might have prescribed inadequate doses of antimicrobial agents in patients with CRRT [5] and unintentionally increase the morbidity and mortality associated with sepsis [6]. The primary aim of drug dosing in this population is to use the loading dose (LD) and adequate maintenance doses to attain pharmacokinetic and pharmacodynamic targets for maximizing antibacterial killing effect and therapeutic outcome [7].

Cefepime dosing recommendations in critically ill patients are based on previously published pharmacokinetic studies [2, 5, 8, 9]. Interestingly, Li and colleagues gathered and analyzed 64 published pharmacokinetic studies in patients receiving CRRT. They revealed that those studies did not completely report key pharmacokinetic parameters to calculate extracorporeal clearance and design drug dosing in patient with CRRT such as type of CRRT modalities, effluent rate, blood flow rate, and extraction coefficient [10]. Some studies used old CRRT techniques or hemofilters and low effluent rates [10]. Neurotoxicity from high cefepime concentrations in patients with reduced renal function has been reported [11–15].

Our study aimed to use the Monte Carlo simulation (MCS) technique to define the proper dosing of cefepime in AKI patients who require CRRT support.

## Methods

### Mathematical pharmacokinetic models

A literature search was performed using the following medical subject heading (MeSH) terms: (1) ‘cefepime’, (2) ‘continuous renal replacement therapy’ or ‘continuous venovenous hemofiltration’ or ‘continuous venovenous hemodialysis’, and (3) ‘pharmacokinetics’ and synonymous words in PubMed. EMBASE and EBSCO were searched with slightly different search terms due to differences of each database. All publications that entered the databases by 31 December 1990 were included. Two investigators (WC and TC) independently identified and evaluated studies for potential inclusion. We restricted our search to articles conducted in adult human subjects and critically ill subjects receiving CRRT. All publications focused on drug pharmacokinetics were gathered. We included only publications that reported all necessary pharmacokinetic parameters for calculation of cefepime dosing regimens. Any disagreement on inclusion was resolved by discussion between the two reviewers. We identified 50 publications, of which 6 were considered relevant and were evaluated [8, 9, 16–19]. All previously published pharmacokinetic studies of cefepime reported only basic pharmacokinetic parameters such as volume of distribution, total drug clearance, non-renal clearance, extraction coefficient, and elimination rate constant [8, 9, 16–19]. In addition, Carlier and colleagues revealed that a one-compartment pharmacokinetic model best fits to describe cefepime characteristics [19]. Consequently, a one-compartment mathematical pharmacokinetic model with first-order elimination of acute kidney disease patients receiving CRRT was developed to predict cefepime disposition in 48 h of the initial therapy. Assuming the patients were anuric, renal clearance applied in the model was 0 mL/min. Previously published cefepime pharmacokinetic parameters in critically ill patients [8, 9, 16–19] and related variability from critically ill patients receiving CRRT were gathered to create models of virtual patients with three modalities. Two thirds of patients in previously published studies were diagnosed as sepsis and septic shock and needed CRRT treatment. Different CRRT settings affect drug dosing [20], and no specific technique of CRRT modality for AKI management is recommended [1]. The commonly used modalities consisted of continuous venovenous hemofiltration (CVVH) with pre-hemofilter dilution techniques, in which replacement fluid is added in blood before going through hemofilter and continuous venovenous hemodialysis (CVVHD) [20]. To construct realistic virtual patients, we added population-specific correlation (*r*^2^) between patient’s body weight, non-renal clearance, and volume of distribution into the models. The lower limit of body weight was set at 40 kg assuming that the virtual patients are adult. In addition, body weights used in the models of virtual patients were obtained from an international database of the International Society of Nephrology (ISN)-funded prospective multicenter observational ongoing study of AKI epidemiology in Southeast Asia entitled The Epidemiology and Prognostic Factors for Mortality in Intensive Care Unit Patients with Acute Kidney Injury in Southeast Asia (SEA-AKI) [21]. It enrolled 6644 critically ill patients from Thailand, Laos, and the Philippines. All described pharmacokinetic parameters are defined in Table 1.

**Table 1:** Parameters used in these models of virtual AKI patients receiving CRRT [14–18]

Pharmacokinetic parameters Mean ± SD (range limits)		
	Hemofiltration (HF)	Hemodialysis (HD)
Weight (kg)	60.72 ± 14.5 (40–230)	
*V*_d_ (L/kg)	0.5 ± 0.23 (0.21–1.11)	
CL_NR_ (mL/min)	24.33 ± 11.25 (13–44)	
Free fraction	0.79 ± 0.09 (0.72–0.85)	
SC or SA	0.79 ± 0.15 (0.47–0.92)	0.77 ± 0.09 (0.65–0.97)

Transmembrane drug clearance was calculated as multiplying effluent flow rate, dialysate (*Q*_d_) and/or ultrafiltrate (*Q*_uf_) flow rate, by extraction coefficient that are sieving coefficient (SC) for hemofiltration and saturation coefficient (SA) for hemodialysis [20]. Total drug clearance was calculated from the summation of non-renal clearance and CRRT clearance. To calculate drug concentration profile in 48 h of initial therapy for evaluation of the probability of target attainment (PTA), elimination rate constant (*k*) was determined by total drug clearance divided by volume of distribution. The *k* value was required to calculate drug concentration at a time. Blood flow rate (*Q*_blood_) for all settings was prescribed as 200 mL/min. The equations used in the models were defined as follows [20]:

CL_HD_ (*L*/*h*) = SA × Q_d_

CL_HF(pre)_ (*L*/*h*) = SC × *Q*_uf_ × [*Q*_plasma_/(*Q*_plasma_ + *Q*_replacement_)]

*k* = (CL_NR_ + CL_HD_)/*V*_d_

*k* = (CL_NR_ + CL_HF_)/*V*_d_

where CL_HF_ is the transmembrane clearance in hemofiltration, *Q*_plasma_ is the plasma flow rate (*Q*_plasma_ = *Q*_blood_ × (1 − hematocrit)), hematocrit is 30%, *Q*_replacement_ is the replacement fluid flow rate (*Q*_replacement_ = *Q*_uf_), CL_HD_ is the transmembrane clearance in hemodialysis, *Q*_d_ is the dialysate flow rate, *k* is the elimination rate constant, CL_NR_ is the non-renal clearance, and *V*_d_ is the volume of distribution.

Effluent rates were prescribed as Kidney Disease: Improving Global Outcomes (KDIGO) recommendation of 20–25 mL/kg/h [1]. A higher effluent rate of 35 mL/kg/h was included in the models to reflect an average common effluent rate used in real-life practice or when high-volume CRRT is needed [22]. Moreover, lower effluent rates of 10–15 mL/kg/h were performed to aid cefepime dosing when low-volume CRRT was prescribed in some situations.

### Cefepime dosing recommendations

Cefepime dosing regimens from available drug dosing recommendations were evaluated in the models. The dosing regimens varied from 1 to 2 g every 12 h to 2 g loading dose followed by 1 g every 8 h or 2 g every 12 h [23–25].

### Monte Carlo simulation and probability of target attainment

Following a previously published method [26, 27], Monte Carlo simulation (Crystal Ball Classroom edition, Oracle) generates drug concentration-time profiles of a group of 5000 virtual patients for each dose to evaluate the PTA. PTA was predicted using pharmacodynamic target of the cumulative percentage of a 48-h period that free cefepime concentration exceeds the minimum inhibitory concentration (MIC) of *Pseudomonas aeruginosa* [28]. Given that microbiological success (eradication or presumed eradication) was significantly associated with the proportion of the dosing interval in which cefepime concentration exceeded four times MIC [29] and the cumulative percentage of free cefepime concentration needed to exceed the MIC, 70% coverage is required to achieve the maximal bactericidal effect [17, 18, 30]. In this study, at least 70% of the cumulative percentage of a 48-h period with four times MIC (70% fT_>4MIC_) and susceptible breakpoint recommended by the Clinical Laboratory Standards Institute (CLSI) [31] for *P. aeruginosa* (8 mg/L) were applied in the models for the first 48 h of initial cefepime therapy. Owing to the differences of the MIC in various health care settings, we also used the MICs of 1, 2, and 4 mg/L in the models to define the optimal dosing regimens for each MIC in the study. The optimal doses were defined as achieving the PTA target of at least 90% of 5000 virtual patients with the lowest daily dose to emphasize cefepime efficacy and consider the risk of toxicity especially neurotoxicity as described below. Different cefepime dosing regimens including recommendations for critically ill patients were evaluated to define the optimal doses.

### Cefepime neurotoxicity

Neurotoxicity of cefepime, defined as confusion, hallucination, convulsion, seizure, and encephalopathy, has been noted in various studies. Most studies in patients with reduced renal function reported cefepime trough concentrations associated with neurotoxicity as an average of 76 (9–224) mg/L [11–15]. We used the concentration of 70 mg/L to be a threshold for expected neurotoxicity that could occur from cefepime in AKI patients receiving CRRT. All cefepime dosing regimens were evaluated for the possibility to develop neurotoxicity at 48-h trough concentration. The optimal doses were required to achieve a previously described target and had the lowest risk of occurring ≥ 70 mg/L of cefepime concentrations in drug concentration-time profiles of 5000 virtual patients for each regimen.

## Results

Characteristics of selected virtual patients who achieve the pharmacodynamic target with the optimal dosing regimens as described in the “Methods” section were compared with input parameters from previously published studies and are shown in Table 2.

**Table 2:** Virtual patient characteristics compared with input pharmacokinetic parameters from published cefepime studies

Pharmacokinetic parameters	Literature-based values (mean ± SD (range limits)) (*N* = 37)	Simulation-based values (mean ± SD (range limits)) (*N* = 5000)
Weight (kg)	60.72 ± 14.5 (40–230)	61.88 ± 13.77 (40.01–142.22)
*V*_d_ (L/kg)	0.5 ± 0.23 (0.21–1.11)	0.49 ± 0.19 (0.21–1.11)
CL_NR_ (mL/min)	24.33 ± 11.25 (13–44)	24.21 ± 7.63 (13.00–43.99)
Free fraction	0.79 ± 0.09 (0.72–0.85)	0.78 ± 0.04 (0.72–0.85)
SC	0.79 ± 0.15 (0.47–0.92)	0.74 ± 0.10 (0.47–0.92)
SA	0.77 ± 0.09 (0.65–0.97)	0.78 ± 0.07 (0.65–0.97)

Table 3 summarizes the PTA results of selected cefepime dosing regimens for treating *P. aeruginosa* using MICs of 1, 2, 4, and 8 on the first 48 h of therapy. The probability of developing neurotoxicity of each regimen of two CRRT modalities and five different effluent rates was presented in Table 4. Applying the aggressive target as CLSI recommended MIC breakpoint of 8 mg/L into the models, all recommended dosing regimens from clinical resources could not attain the targets with two different modalities. Considering efficacy from the PTA target and the probability of developing neurotoxicity, the regimen of 2 g loading dose followed by 1.5–1.75 g every 8 h achieved the aforementioned targets of > 90% for all CRRT settings with KDIGO recommended effluent rates in a range of 20–25 mL/kg/h (Table 5). In addition, the probability of neurotoxicity occurred when cefepime concentrations > 70 mg/L at 48 h was approximately 0.06–17% (Table 4). The PTA of cefepime regimens according to various MICs, effluent rates, and CRRT modalities is presented in Table 3. The recommendations of cefepime regimens for critically ill patients receiving three different CRRT modalities, effluent rates, and various MICs are shown in Table 5.

**Table 3:** PTAs of all recommended cefepime dosing regimens for Gram-negative infections in two CRRT modalities with different effluent rates and various MICs

Cefepime dosing regimen	Pre-dilution CVVH					CVVHD				
	Effluent rates (mL/kg/h)	PTA (%)				Effluent rates (mL/kg/h)	PTA (%)			
		MIC (mg/L)					MIC (mg/L)			
		1	2	4	8		1	2	4	8
250 mg Q8H	10	98.62	53.10	0.22	0.00	10	98.64	49.90	0.04	0.00
	15	98.50	43.78	0.02	0.00	15	98.46	40.86	0.00	0.00
	20	98.02	36.10	0.00	0.00	20	97.68	29.3	0.00	0.00
	25	98.12	27.06	0.00	0.00	25	96.52	16.58	0.00	0.00
	35	96.42	13.72	0.00	0.00	35	91.62	3.52	0.00	0.00
750 mg LD then 500 mg Q12H	10	100	95.24	20.88	0.00	10	99.98	94.42	17.96	0.00
	15	100	91.44	11.00	0.00	15	99.98	89.06	8.12	0.00
	20	99.96	86.86	5.12	0.00	20	99.88	80.80	1.54	0.00
	25	99.80	81.28	1.64	0.00	25	99.52	71.46	0.14	0.00
	35	99.38	66.48	0.10	0.00	35	98.72	42.06	0.00	0.00
1 g LD then 500 mg Q12H	10	100	98.42	38.36	0.00	10	100	98.02	33.30	0.00
	15	99.96	96.88	25.34	0.00	15	99.98	95.60	18.52	0.00
	20	99.96	94.00	14.22	0.00	20	99.86	90.26	7.24	0.00
	25	99.90	90.24	6.86	0.00	25	99.84	83.04	1.96	0.00
	35	99.74	80.30	1.00	0.00	35	99.08	58.44	0.04	0.00
750 mg Q12H	10	100	99.30	51.16	0.06	10	100	99.18	47.34	0.00
	15	100	98.70	38.72	0.00	15	100	98.36	33.6	0.00
	20	100	97.98	29.42	0.00	20	100	96.66	20.50	0.00
	25	99.98	96.78	18.30	0.00	25	99.94	93.58	8.90	0.00
	35	99.86	91.54	6.72	0.00	35	99.76	80.52	0.98	0.00
1 g Q12H	10	100	99.96	83.58	6.30	10	100	100	82.72	5.52
	15	100	99.92	76.62	2.42	15	100	99.86	72.94	1.12
	20	100	99.82	68.78	0.62	20	100	99.52	61.14	0.06
	25	100	99.52	61.18	0.10	25	100	99.00	47.26	0.00
	35	99.98	98.78	42.26	0.00	35	99.94	96.74	19.50	0.00
750 mg Q8H	10	100	99.98	92.48	19.38	10	100	99.98	92.08	16.34
	15	100	99.98	90.78	12.92	15	100	100	87.92	8.86
	20	100	99.94	86.92	6.56	20	100	99.96	83.70	3.14
	25	100	99.98	83.92	0.26	25	100	99.96	77.50	0.56
	35	100	99.98	73.96	0.52	35	100	99.88	54.20	0.00
1 g LD then 750 mg Q8H	10	100	100	96.12	28.10	10	100	100	95.98	26.90
	15	100	100	94.92	18.86	15	100	100	94.26	14.62
	20	100	100	93.08	11.82	20	100	99.98	91.14	7.50
	25	100	100	90.62	7.00	25	100	100	84.68	1.98
	35	100	99.98	82.84	1.30	35	100	99.92	66.64	0.02
1 g Q8H	10	100	100	98.58	54.02	10	100	100	98.60	51.60
	15	100	100	98.20	45.68	15	100	100	98.14	40.96
	20	100	100	97.96	35.56	20	100	100	97.54	29.68
	25	100	100	97.96	29.46	25	100	100	96.50	18.22
	35	100	100	96.56	13.88	35	100	100	91.62	3.58
1.75 g then 1.5 g Q 8 H	10	100	100	100	94.38	10	100	100	100	94.28
	15	100	100	100	92.44	15	100	100	99.98	91.72
	20	100	100	100	90.04	20	100	100	99.98	88.04
	25	100	100	99.96	87.46	25	100	100	99.92	79.64
	35	100	100	99.96	78.62	35	100	100	99.98	61.84
2 g LD then 1.5 g Q8H	10	100	100	100	96.04	10	100	100	100	95.3
	15	100	100	100	95.24	15	100	100	100	94.36
	20	100	100	99.98	93.26	20	100	100	99.98	90.66
	25	100	100	100	90.50	25	100	100	99.98	84.60
	35	100	100	100	82.96	35	100	100	99.96	66.70
1.75 g Q8H	10	100	100	100	96.94	10	100	100	100	96.22
	15	100	100	100	96.34	15	100	100	100	95.70
	20	100	100	100	95.42	20	100	100	100	94.64
	25	100	100	100	94.58	25	100	100	100	92.00
	35	100	100	100	89.48	35	100	100	99.98	77.76
2 g LD then 1.75 g Q8H	10	100	100	100	97.80	10	100	100	100	97.44
	15	100	100	100	97.36	15	100	100	99.98	96.60
	20	100	100	100	96.70	20	100	100	100	95.46
	25	100	100	99.98	95.90	25	100	100	100	93.20
	35	100	100	100	92.52	35	100	100	99.98	82.22
2 g Q8H	10	100	100	100	98.60	10	100	100	100	98.36
	15	100	100	100	98.32	15	100	100	100	98.18
	20	100	100	100	98.44	20	100	100	100	97.54
	25	100	100	100	98.00	25	100	100	100	97.02
	35	100	100	100	96.66	35	100	100	100	91.24

*PTA* probability of target attainment, *CVVH* continuous venovenous hemofiltration, *CVVHD* continuous venovenous hemodialysis, *LD* loading dose

**Table 4:** The probability of developing neurotoxicity from selected cefepime dosing regimens

Cefepime dosing regimens	Pre-dilution CVVH		CVVHD	
	Effluent rate (mL/kg/h)	48-h trough probability ≥ 70 mg/L (%)	Effluent rate (mL/kg/h)	48-h trough probability ≥ 70 mg/L (%)
1 g Q8H	10	0.18	10	0.04
	15	0.00	15	0.00
	20	0.00	20	0.00
	25	0.00	25	0.00
	35	0.00	35	0.00
1.75 g LD then 1.5 g Q8H	10	26.28	10	22.30
	15	11.54	15	7.02
	20	4.46	20	1.44
	25	1.26	25	0.08
	35	0.14	35	0.00
2 g LD then 1.5 g Q8H	10	26.04	10	21.7
	15	13.36	15	7.40
	20	5.12	20	0.98
	25	1.50	25	0.06
	35	0.08	35	0.00
1.75 g Q8H	10	45.44	10	41.30
	15	29.14	15	22.24
	20	16.84	20	7.82
	25	7.96	25	1.94
	35	1.22	35	0.04
2 g LD then 1.75 g Q8H	10	45.12	10	42.10
	15	32.46	15	22.30
	20	17.16	20	7.78
	25	8.04	25	1.76
	35	1.38	35	0.04
2 g Q8H	10	61.04	10	58.10
	15	47.86	15	40.52
	20	33.48	20	20.84
	25	20.90	25	8.98
	35	6.52	35	0.26

*CVVH* continuous venovenous hemofiltration, *CVVHD* continuous venovenous hemodialysis, *LD* loading dose

**Table 5:** Recommendations of cefepime dosing regimens for treating *P. aeruginosa* infections with various MICs in critically ill patients receiving CRRT

Actual MIC (mg/L)	Target MIC* (mg/L)	Effluent rates (mL/kg/h)	CVVH (pre-hemofilter dilution)	CVVHD
1	4	10–15	250 mg Q8H	250 mg Q8H
		20–25	250 mg Q8H	250 mg Q8H
		35	250 mg Q8H	250 mg Q8H
2	8	10–15	750 mg LD then 500 mg Q12H	1 g LD then 500 mg Q12H
		20–25	1 g LD then 500 mg Q12H	750 mg Q12H
		35	750 mg Q12H	1 g Q12H
4	16	10–15	750 mg Q8H	1 g LD then 750 mg Q8H
		20–25	1 g LD then 750 mg Q8H	1 g Q8H
		35	1 g Q8H	1 g Q8H
8	32	10–15	1.75 g LD then 1.5 g Q8H	1.75 g LD then 1.5 g Q8H
		20–25	2 g LD then 1.5 g Q8H	1.75 g Q8H
		35	2 g LD then 1.75 g Q8H	2 g Q8H

*CVVH* continuous venovenous hemofiltration, *CVVHD* continuous venovenous hemodialysis, *LD* loading dose

*Pharmacodynamic target defined as at least 70% of the cumulative percentage of a 48-h period with four times MIC (70% fT_>4MIC_)

**Fig. 1:**
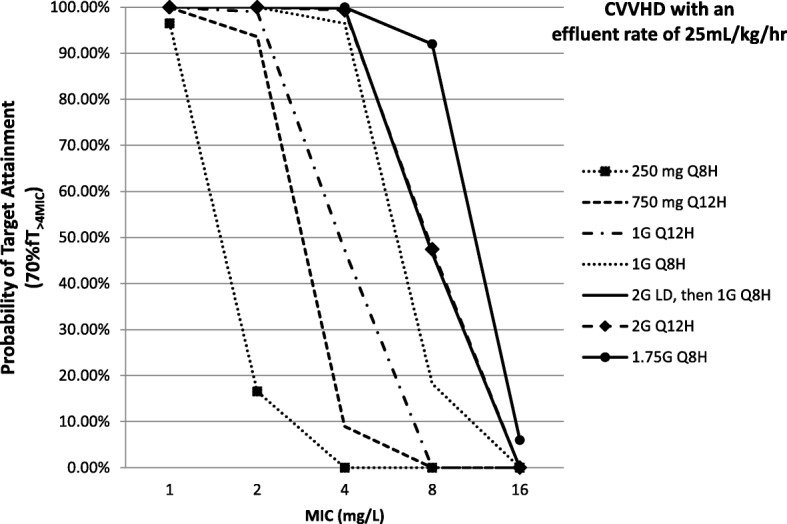
PTA results of cefepime dosing regimens at different MICs in CVVHD and 25 mL/kg/h effluent rate for management of Gram-negative infections caused by *P. aeruginosa* (> 70% fT_>4MIC_; MIC of 8 mg/L) in virtual patients for the first 48 h

If greater effluent rates such as 35 mL/kg/h are required, the cefepime dosing regimen for *P. aeruginosa* infection (MIC of 8 mg/L) using the aggressive pharmacodynamic target was 2 g LD followed by 1.75–2 g every 8 h with a higher risk of cefepime-induced neurotoxicity (≤ 33%) (Tables 4 and 5). When CRRT with lower effluent rates of 10–15 mL/kg/h was prescribed, the cefepime dosing regimen of 1.75 g loading dose followed by 1.5 g every 8 h was needed to achieve the aggressive target for *P. aeruginosa* infection (MIC of 8 mg/L) (Table 4). Figure 1 illustrates the PTA at 70% fT_>4MIC_ (MIC of 8 mg/L) for selected cefepime dosing regimens of CVVHD with an effluent rate of 25 mL/kg/h.

## Discussion

This is the first simulation study applying MCS technique to evaluate cefepime dosing regimens for management of *P. aeruginosa* infection in critically ill patients. Pharmacokinetic parameters collected from previously published studies [8, 9, 16–19], body weights as described in the “Methods” section, and CRRT settings with five different effluent rates (10, 15, 20, 25, and 35 mL/kg/h) [1, 22] were incorporated into the models to predict cefepime disposition in critically ill patients receiving CRRT for 48 h. Moreover, correlations between used pharmacokinetic parameters were applied in the models to create population-specific virtual patients. As shown in Table 2, this study showed that MCS technique created virtual patient pharmacokinetics that were similar to which parameters gathered from previous studies. This technique therefore could be an effective tool to build realistic patients and guide drug dosing regimens in various groups of patients, especially this population.

The pharmacodynamic target of 70% fT_>4MIC_ was associated with maximum bactericidal effects [17, 18, 30]. Given the results from Tam and colleagues, bactericidal activity of cefepime is optimized at concentrations approximately four times MIC [29]. We decided to apply aggressive target as 70% fT_>4MIC_ in the models as aforementioned in the “Methods” section. However, using the aggressive target could lead to excessive drug dosages with the risk of cefepime-induced neurotoxicity. Clinical monitoring of cefepime adverse reactions should be concerned.

This study revealed that the regimen of 2 g loading dose followed by 1.5–1.75 g every 8 h achieved the PTA target for *P. aeruginosa* (MIC of 8 mg/L) with two different modalities in ≥ 90% of virtual patients receiving CRRT with KDIGO recommended effluent rate of 20–25 mL/kg/h. The expected neurotoxicity risk occurred with the suggested regimen from our simulations were in a range of 0.06–17% according to the effluent rates and CRRT modalities (Table 4). Clinical monitoring of cefepime-induced neurotoxicity is needed when the recommended cefepime dosing regimen is prescribed. Notably, no clinical recommended regimens exceeded the PTA target of *P. aeruginosa*. It was aligned with the results from Seyler et al. that they used the pharmacodynamic target of 70% fT_>4MIC_ (8 mg/L) which was 32 mg/L as we applied in this study for *P. aeruginosa*. They revealed that the recommended doses of cefepime could not achieve the target for critically ill patients with CRRT for the first 48 h (0% PTA) [18]. Moreover, dosing regimens for *P. aeruginosa* infection were different depending on MICs used in the simulations (Table 5). It explained that cefepime dosing regimens were associated with local MIC values in each setting.

The pharmacokinetic changes of hydrophilic drugs in critically ill patients such as increased volume of distribution due to fluid accumulation, decreased protein binding and metabolism can cause lower drug concentrations especially when conventional dosing regimens were used [5]. The cefepime volume of distribution gathered from critically ill patients and used in this study was approximately 30 L (0.5 ± 0.23 L/kg). The value was larger than that reported in normal population (4–20 L) [2–4]. As volume of distribution is taken into account in a mathematical equation of drug clearance as CL = *k* × *V*_d_, it affects drug clearance and the probability of target attainment when volume of distribution increases. Given that AKI patients may have well-preserved non-renal drug clearance [32], an average non-renal clearance gathered from previously published studies (24.33 ± 11.25 mL/min) was similar to the values reported from healthy volunteers and patients with renal insufficiency in a range of 10–30 mL/min [3, 4, 33]. Additionally, hypoalbuminemia occurred in ICU patients was reported in a range of 40–50% [34] and could increase free drug concentrations that would be removed by CRRT, the liver, and the kidney. Given these reasons described earlier, the loading dose concept is very crucial to attain the PTA target in these situations.

An effluent rate contributes to extracorporeal clearance defined by the described equation. Higher effluent rate requires a higher dose to compete the PTA target in the population. When CRRT setting was prescribed with an effluent rate of 35 mL/kg/h, cefepime doses would be 2 g LD followed by 1.75–2 g every 8 h for *P. aeruginosa* (MIC of 8 mg/L) to achieve the PTA target. Undoubtedly, if the lower effluent rates of 10–15 mL/kg/h were utilized, the lower cefepime loading dose of 1.75 g was suggested with same maintenance doses as compared with using KDIGO-recommended effluent rates of 20–25 mL/kg/h (Table 5).

Some drugs can be removed by membrane interaction known as the adsorption phenomenon. Although the clinical significance has not been evaluated, CRRT hemofilter types do not significantly affect extracorporeal drug clearance and selection of drug dosing regimen due to early saturation of this phenomenon [20].

Owing to the assumption of MCS that generates only adult patients using pharmacokinetic parameters from previously published studies and ICU patient’s body weights, those recommendations of cefepime should be applied for only patients who match our assumption such as anuric patients, same effluent flow rates. Another limitation of our study is the MIC breakpoint from the Clinical Laboratory Standards Institute [31] used in the models. This value of 8 mg/L in the study implies a worst situation of when a susceptible *P. aeruginosa* for cefepime is reported. The dosing recommendations therefore would be adjusted for the settings that have lower reported MICs as shown in Table 5 and Fig. 1. In addition, Su and colleagues conducted a hospital-based retrospective study in 90 hospitalized patients. The results showed that the survival rate of patients with a positive blood culture for susceptible *P. aeruginosa* receiving cefepime as the primary therapy was significantly lower in a group with a higher MIC (> 4 mg/L) compared with that in the lower MIC group (< 4 mg/L) (72.6% vs 23.5%, *p* < 0.0001) [35]. Consequently, we suggested to dose cefepime based on MIC values of each setting (Table 5). An alternative therapy might be considered when a patient who has *P. aeruginosa* infection with a cefepime MIC of > 4 mg/L was identified.

Clinical validation of these results is warranted. Reconsidering using these regimens from clinically available resources in critically ill patients would be suggested, and close monitoring of efficacy when prescribing the conventional dosing regimen is very important.

## Conclusion

The MCS technique can be a valuable tool for evaluating drug dosing in critically ill patients receiving CRRT when limited pharmacokinetic data is a major concern. These results revealed that the optimal doses for critically ill patient receiving CRRT were higher than recommended doses form clinical available resources for treating *P. aeruginosa*. The dosing regimen of 2 g LD was followed by 1500–1750 mg every 8 h for critically ill patients receiving CRRT with KDIGO-recommended effluent rates. If the higher effluent rate is prescribed, drug doses should be increased. The MIC values of each setting were an important factor to design cefepime dosing regimens. Clinical study is absolutely needed to validate our recommendations.

## Abbreviations

AKI: Acute kidney injury
CL: Clearance
CL_HD_: Transmembrane clearance
CL_HF_: Transmembrane clearance
CL_NR_: Non-renal clearance
CLSI: Clinical Laboratory Standards Institute
CRRT: Continuous renal replacement therapy
CVVH: Continuous venovenous hemofiltration
CVVHD: Continuous venovenous hemodialysis
fT_>4MIC_: The cumulative percentage of a 48-h period with four times MIC
g: Gram
h: Hour
ISN: International Society of Nephrology
k: Elimination rate constant
KDIGO: Kidney Disease: Improving Global Outcomes
kg: Kilogram
L: Liter
LD: Loading dose
MCS: Monte Carlo simulations
mg: Milligram
MIC: Minimum inhibitory concentration
mL: Milliliter
*N*: Number
PTA: Probability of target attainment
q: Every
*Q*_blood_: Blood flow rate
*Q*_d_: Dialysate flow rate
*Q*_plasma_: Plasma flow rate
*Q*_replacement_: Replacement fluid flow rate
*Q*_uf_: Ultrafiltrate flow rate
*r*^2^: Population-specific correlation
SA: Saturation coefficient
SC: Sieving coefficient
SD: Standard deviation
SEA-AKI: Southeast Asia entitled The Epidemiology and Prognostic Factors for Mortality in Intensive Care Unit Patients with Acute Kidney Injury in Southeast Asia
*V*_d_: Volume of distribution
